# Guidance for Implementing COVID-19 Prevention Strategies in the Context of Varying Community Transmission Levels and Vaccination Coverage

**DOI:** 10.15585/mmwr.mm7030e2

**Published:** 2021-07-27

**Authors:** Athalia Christie, John T. Brooks, Lauri A. Hicks, Erin K. Sauber-Schatz, Jonathan S. Yoder, Margaret A. Honein, CDC COVID-19 Response Team

**Affiliations:** 1 CDC COVID-19 Response Team.

COVID-19 vaccination remains the most effective means to achieve control of the pandemic. In the United States, COVID-19 cases and deaths have markedly declined since their peak in early January 2021, due in part to increased vaccination coverage (*1*). However, during June 19–July 23, 2021, COVID-19 cases increased approximately 300% nationally, followed by increases in hospitalizations and deaths, driven by the highly transmissible B.1.617.2 (Delta) variant* of SARS-CoV-2, the virus that causes COVID-19. Available data indicate that the vaccines authorized in the United States (Pfizer-BioNTech, Moderna, and Janssen [Johnson & Johnson]) offer high levels of protection against severe illness and death from infection with the Delta variant and other currently circulating variants of the virus (*2*). Despite widespread availability, vaccine uptake has slowed nationally with wide variation in coverage by state (range = 33.9%–67.2%) and by county (range = 8.8%–89.0%).^†^ Unvaccinated persons, as well as persons with certain immunocompromising conditions (*3*), remain at substantial risk for infection, severe illness, and death, especially in areas where the level of SARS-CoV-2 community transmission is high. The Delta variant is more than two times as transmissible as the original strains circulating at the start of the pandemic and is causing large, rapid increases in infections, which could compromise the capacity of some local and regional health care systems to provide medical care for the communities they serve. Until vaccination coverage is high and community transmission is low, public health practitioners, as well as schools, businesses, and institutions (organizations) need to regularly assess the need for prevention strategies to avoid stressing health care capacity and imperiling adequate care for both COVID-19 and other non–COVID-19 conditions. CDC recommends five critical factors be considered to inform local decision-making: 1) level of SARS-CoV-2 community transmission; 2) health system capacity; 3) COVID-19 vaccination coverage; 4) capacity for early detection of increases in COVID-19 cases; and 5) populations at increased risk for severe outcomes from COVID-19. Among strategies to prevent COVID-19, CDC recommends all unvaccinated persons wear masks in public indoor settings. Based on emerging evidence on the Delta variant (*2*), CDC also recommends that fully vaccinated persons wear masks in public indoor settings in areas of substantial or high transmission. Fully vaccinated persons might consider wearing a mask in public indoor settings, regardless of transmission level, if they or someone in their household is immunocompromised or is at increased risk for severe disease, or if someone in their household is unvaccinated (including children aged <12 years who are currently ineligible for vaccination).

The principal mode by which persons are infected with SARS-CoV-2 is through exposure to respiratory fluids carrying infectious virus.^§^ The risk for SARS-CoV-2 transmission in outdoor settings is low (*4*,*5*). CDC recommends that public health practitioners and organizations prioritize prevention strategies for indoor settings. No one strategy is sufficient to prevent transmission, and multiple interventions should be used concurrently to reduce the spread of disease (*6*). Proven effective strategies against SARS-CoV-2 transmission, beyond vaccination, include using masks consistently and correctly (*7*,*8*), maximizing ventilation both through dilution (*9*,*10*) and filtration (*11*) of air, and maintaining physical distance and avoiding crowds (*12*,*13*). Basic public health measures such as staying home when sick, handwashing, and regular cleaning of high-touch surfaces should also be encouraged.

## Level of SARS-CoV-2 Community Transmission

A person’s risk for SARS-CoV-2 infection is directly related to the risk for exposure to infectious persons, which is largely determined by the extent of SARS-CoV-2 circulation in the surrounding community.^¶^ CDC recommends assessing the level of community transmission using, at a minimum, two metrics: new COVID-19 cases per 100,000 persons in the last 7 days and percentage of positive SARS-CoV-2 diagnostic nucleic acid amplification tests in the last 7 days. For each of these metrics, CDC classifies transmission values as low, moderate, substantial, or high (Table). If the values for each of these two metrics differ (e.g., one indicates moderate and the other low), then the higher of the two should be used for decision-making. CDC recommends the geographic unit of analysis be county or core-based statistical area. In rural areas with low population densities, multiple counties might need to be combined to increase available data so that reliable inferences can be made. The level of SARS-CoV-2 transmission for any given area can change rapidly and should be reassessed at least weekly to ensure that the necessary layered prevention strategies are in place. In areas of substantial or high transmission, CDC recommends community leaders encourage vaccination and universal masking in indoor public spaces in addition to other layered prevention strategies to prevent further spread. Updated community transmission levels, as well as other indicators related to COVID-19, are available by county and state at the online CDC COVID Data Tracker** and are already used by many public health departments.

**TABLE:** CDC core indicators of and thresholds for community transmission levels of SARS-CoV-2

Indicator	Transmission level			
	Low	Moderate	Substantial	High
New cases per 100,000 persons in the past 7 days*	0–9.99	10.00–49.99	50.00–99.99	≥100.00
Percentage of positive nucleic acid amplification tests in the past 7 days^†^	<5.00	5.00–7.99	8.00–9.99	≥10.00

* Number of new cases in the county (or other administrative level) in the past 7 days divided by the population in the county (or other administrative level) multiplied by 100,000.

^†^ Number of positive tests in the county (or other administrative level) during the past 7 days divided by the total number of tests performed in the county (or other administrative level) during the past 7 days. https://www.cdc.gov/coronavirus/2019-ncov/lab/resources/calculating-percent-positivity.html

## Health System Capacity

Data on usage of clinical care resources to manage patients with COVID-19 reflect underlying community disease incidence and can signal when urgent implementation of layered prevention strategies might be necessary to prevent overloading the health care system. Strains on critical care capacity can increase COVID-19 mortality (*14*,*15*) while decreasing the availability and use of health care resources for non–COVID-19 related medical care (*16*,*17*). CDC recommends public health departments and health care institutions monitor the available number and fraction of staffed inpatient and intensive care unit beds and develop thresholds, based on local health care system usage and remaining capacity, that would trigger community-wide application of layered prevention strategies.

## COVID-19 Vaccination Coverage

Monitoring vaccination coverage in communities and organizations is recommended by CDC to gauge progress, focus vaccination efforts on populations whose coverage is low, and inform the need for additional prevention strategies. As of July 23, 2021, the proportion of the total U.S. population who is fully vaccinated is 48.9%.^††^ Of the 2,945 (91.4%) U.S. counties reporting, vaccination coverage is <40% in 1,856 (63.0%) and 40%–49.9% in 672 (22.8%); only 417 (14.2%) of counties reported ≥50% COVID-19 vaccination coverage. Primary vaccination efforts should be accelerated in counties with low vaccination coverage. Public health practitioners should work with clinicians and community partners to build confidence in the vaccine and ensure equitable access.^§§^ Organizations should establish supportive policies, such as allowing workers to receive vaccines during work hours or to take paid leave to get vaccinated at a community site, as well as offering flexible, nonpunitive sick leave options for employees.

## Capacity for Early Detection of Increases in COVID-19 Cases

Certain populations are at high risk for exposure to, and thereby infection with, SARS-CoV-2. Such populations are especially well-suited for sentinel monitoring efforts to detect the early introduction and spread of COVID-19, particularly in areas with low vaccination coverage or where layered prevention strategies are not in use. CDC considers the capacity to monitor COVID-19 incidence in the following populations particularly useful due to their high risk of exposure or severe illness: students and staff members of kindergarten–grade 12 schools and institutions of higher education, health care workers, residents and staff members of long-term care facilities, incarcerated persons, homeless persons, and workers in high-density work sites (*18*–*23*). 

Serial screening testing is an effective method to monitor for the early introduction and spread of COVID-19 (*6*). Low case detection rates can help demonstrate the effectiveness of current prevention strategies, thereby reducing barriers for returning to in-person learning and work. Rising case detection rates can serve as an early warning signal that prevention strategies need to be strengthened or added in the facility and the broader community. In addition, strategic serial testing can help stop transmission by rapidly identifying asymptomatic cases, which are estimated to be the source for at least 50% of SARS-CoV-2 transmission (*24*,*25*). With rapid identification, infectious persons can be isolated and contact tracing and quarantine can be promptly initiated to control further SARS-CoV-2 transmission.

## Populations at Risk for Severe Outcomes from COVID-19

CDC recommends additional prevention strategies to safeguard populations at highest risk for severe outcomes from COVID-19, particularly in the context of the highly transmissible Delta variant. Unvaccinated persons remain at risk for infection, severe illness, and death. Advanced age, pregnancy, and an increasingly well-defined set of underlying medical conditions increase the risk for serious outcomes from COVID-19 among unvaccinated persons.^¶¶^ In addition, long-standing systemic health and social inequities have put members of certain racial and ethnic minority groups at increased risk for serious illness and mortality from COVID-19. Persons taking immunosuppressive medications, persons with hematologic cancers, and hemodialysis patients, among others, have shown reduced immunologic responses to COVID-19 mRNA vaccination and might remain at increased risk for severe COVID-19 following vaccination (*3*). CDC recommends unvaccinated persons should continue following all prevention strategies, including wearing a mask, until they are fully vaccinated. Immunocompromised persons should continue to take all recommended precautions until advised otherwise by their health care provider. Although COVID-19 vaccines authorized in the United States remain effective against severe outcomes from SARS-CoV-2 infection, a small proportion of persons who are fully vaccinated may become infected. Emerging evidence suggests that fully vaccinated persons who do become infected with the Delta variant are at risk for transmitting it to others (*2*), (CDC COVID-19 Response Team, unpublished data, 2021); therefore, CDC also recommends that fully vaccinated persons wear a mask in public indoor settings in areas of substantial or high transmission, and consider wearing a mask regardless of transmission level if they or someone in their household is immunocompromised or at increased risk for severe disease, or if someone in their household is unvaccinated (including children aged <12 years who are currently ineligible for vaccination). Public health practitioners and organizations should consider the characteristics of their local or setting-specific populations when determining whether to strengthen or add layered prevention strategies not only for effective disease control, but also to protect those persons at greatest risk for severe illness or death.

## Discussion

The most important public health action to end the pandemic remains increasing vaccination coverage, which saves lives, prevents illness, and reduces the spread of COVID-19. Effective COVID-19 prevention strategies are well documented and can help reduce community transmission until high vaccination coverage is achieved (*6*). To maximize protection of the community, prevention strategies should be strengthened or added if transmission worsens. Prevention strategies should only be relaxed or lifted after several weeks of continuous sustained improvement in the level of community transmission. In areas with low or no SARS-CoV-2 transmission and with testing capacity in place to detect early introduction or increases in spread of the virus, layered prevention strategies might be removed one at a time while monitoring closely for any evidence that COVID-19 cases are increasing.

The widespread availability and administration of COVID-19 vaccines has changed the trajectory of the pandemic in the United States and significantly reduced hospitalization and mortality among vaccinated persons (*1*). Increasing the proportion of eligible persons who are vaccinated reduces the risk for substantial or high community-wide transmission, which in turn reduces the risk for the emergence of new variants that could have the potential to overcome vaccine-induced immunity. However, vaccination coverage varies across the United States, and transmission risk remains considerable in areas with low vaccination coverage. Decisions to add or remove effective prevention strategies should be based on local data and public health recommendations. The emergence of more transmissible SARS-CoV-2 variants, including Delta, increases the urgency to expand vaccination coverage and for public health agencies and other organizations to collaboratively monitor the status of the pandemic in their communities and continue to apply layered prevention strategies to minimize preventable illness and death.

**Summary**

**What is already known about this topic?**

COVID-19 vaccines authorized in the United States are effective against severe illness and death from SARS-CoV-2 infection; however, current U.S. coverage is uneven. Implementation of layered prevention strategies reduces SARS-CoV-2 transmission.

**What is added by this report?**

Given the spread of the highly transmissible Delta variant, local decision-makers should assess the following factors to inform the need for layered prevention strategies across a range of settings: level of SARS-CoV-2 community transmission, health system capacity, vaccination coverage, capacity for early detection of increases in COVID-19 cases, and populations at risk for severe outcomes from COVID-19.

**What are the implications for public health practice?**

Although increasing COVID-19 vaccination coverage remains the most effective means to achieve control of the pandemic, additional layered prevention strategies will be needed in the short-term to minimize preventable morbidity and mortality.

## Footnotes

* Point-in-time information is available from CDC COVID Data Tracker. https://covid.cdc.gov/covid-data-tracker/#variant-proportions

† Persons are considered fully vaccinated if ≥2 weeks have elapsed following receipt of the second dose in a 2-dose series of Moderna or Pfizer-BioNTech mRNA COVID-19 vaccine, or ≥2 weeks following receipt of 1-dose of Janssen (Johnson & Johnson) vaccine. Data are available from CDC COVID Data Tracker. https://covid.cdc.gov/covid-data-tracker

§
https://www.cdc.gov/coronavirus/2019-ncov/science/science-briefs/sars-cov-2-transmission.html

¶
https://www.cdc.gov/coronavirus/2019-ncov/science/science-briefs/transmission_k_12_schools.html

**
https://covid.cdc.gov/covid-data-tracker/#county-view

†† CDC’s COVID Data Tracker accessed July 23, 2021. https://covid.cdc.gov/covid-data-tracker/#vaccinations

§§
https://www.cdc.gov/vaccines/covid-19/vaccinate-with-confidence.html

¶¶
https://www.cdc.gov/coronavirus/2019-ncov/science/science-briefs/underlying-evidence-table.html
